# The incredible vulnerability that reproduction poses for plant species in a warming world

**DOI:** 10.1111/nph.71422

**Published:** 2026-07-10

**Authors:** Derek A. Denney, Annabelle L. Taylor, Emily B. Josephs, John H. Willis, David B. Lowry

**Affiliations:** ^1^ Plant Resilience Institute Michigan State University East Lansing 48824 MI USA; ^2^ Department of Plant Biology Michigan State University East Lansing 48824 MI USA; ^3^ Ecology, Evolution, and Behavior Program Michigan State University East Lansing 48824 MI USA; ^4^ Department of Biology Duke University Durham 27708 NC USA

**Keywords:** fitness, heat stress, pollen development, reproduction, seed filling

## Abstract

Temperatures are rising globally and threatening the persistence of natural plant populations. Elevated temperatures disrupt gametogenesis, fertilization, and seed filling, often at lower thresholds than those affecting photosynthesis, growth, or survival. While crop scientists have found that key reproductive stages are particularly vulnerable to heat stress across plant systems, ecological and evolutionary studies have largely focused on other fitness metrics to estimate populations' resilience to warming. We advocate for integrating pollen, ovule, and seed filling developmental metrics into ecological and evolutionary studies to improve predictions of plant population dynamics under future climates. Such studies will offer not only a better understanding of how natural populations will respond to increasing temperature stress but also are likely to reveal novel mechanistic insights that can be utilized to improve crop resilience in a warming world.

## Graphical abstract

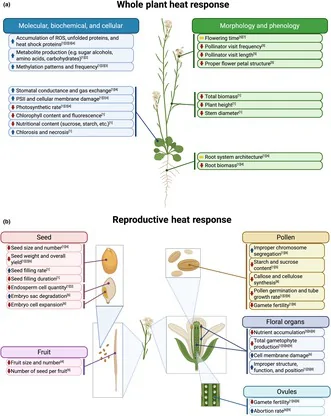

## Introduction

Anthropogenic climate change is causing elevated temperatures and increasingly severe heat waves, pushing natural populations to their physiological, reproductive, and demographic limits (Parmesan, 2006; Parmesan & Hanley, 2015; Arnold *et al*., 2026). While ecological and evolutionary biologists have long appreciated the consequences of climate change, the underlying mechanisms of how and why populations will be impacted by heat are still largely unexplored in natural plant populations (Haider *et al*., 2021).

It is widely recognized that temperatures above a critical threshold can cause irreversible physiological damage to plants (Jagadish *et al*., 2021). However, less appreciated is that reproductive failure can occur at much lower temperatures. While plants can persist and maintain vegetative growth under heat stress, even for brief exposure to extremely high temperatures (approaching 60°C in some species), the average optimal temperatures for gametophyte and seed development are *c*. 26°C in crops and horticultural plants (Nievola *et al*., 2017; Tushabe & Rosbakh, 2025), indicating a mismatch between thermal tolerances in growth vs reproduction. This mismatch reflects the heightened vulnerability of sensitive life‐history stages, particularly early development and reproduction, when even brief heat stress can disrupt key processes, thereby reducing reproductive success and recruitment (Arnold *et al*., 2026). Crop scientists have recognized that the development of plant reproductive tissues – especially pollen, ovules, and seed development – is generally the most vulnerable to rising global temperatures (Zinn *et al*., 2010; Chaturvedi *et al*., 2021). Consequently, extensive research efforts have been made to understand the mechanisms underlying reproductive failure under heat stress in agricultural systems (Table 1).

**Table 1:** Effects of elevated temperature and heat stress on plants: this table provides a nonexhaustive list of recent reviews summarizing the mechanisms and effects of elevated temperature and heat stress on plant reproductive development in crop species.

Structure	References
Whole plant	Datta *et al*. (2024); Haider *et al*. (2021); Jagadish *et al*. (2021); Kan *et al*. (2023); Zhang *et al*. (2021)
Whole flower	Ali & Muday (2024); Lohani *et al*. (2020); Resentini *et al*. (2023); Sinha *et al*. (2021); van der Kooi *et al*. (2019); Walsh *et al*. (2019); Walters *et al*. (2022)
Pollen	Althiab‐Almasaud *et al*. (2024); Chaturvedi *et al*. (2021); Liu *et al*. (2021); Lohani *et al*. (2025); Mesihovic *et al*. (2016)
Pistil	Wang *et al*. (2021); Resentini *et al*. (2023)

In contrast to crop studies, many ecological and evolutionary studies have not distinguished between the effects of heat stress on vegetative growth and reproduction. These studies often focus on proxies of an individual's fitness, using traits such as biomass, survival, and flower or fruit number (Wadgymar *et al*., 2024). While useful and appropriate, these proxies may not capture the full effects of elevated temperatures on reproduction. Although some recent plant evolutionary ecology studies have recognized heat impacts on plant reproductive development (Heiling & Koski, 2024; Tushabe *et al*., 2025), important complementary insights from the crop literature remain underutilized in ecological and evolutionary contexts.

Here, we highlight the effects of heat on pollen and seed development and the reproductive consequences of whole‐plant heat response mechanisms, with a particular focus on herbaceous angiosperms. Our goal is to inspire scientists to incorporate the susceptibility of plant reproduction to heat into future ecological, evolutionary and conservation research. Developing effective strategies to mitigate climate change will require clarifying the underlying mechanisms of extinction due to warmer temperatures. Moreover, future studies of genetic variation in reproductive heat tolerance within natural plant populations are likely to inform crop science by revealing new mechanisms that enhance reproductive resilience to elevated temperatures (Yeaman, 2025).

## Plant reproductive development is disrupted by exposure to heat

**Fig. 1:**
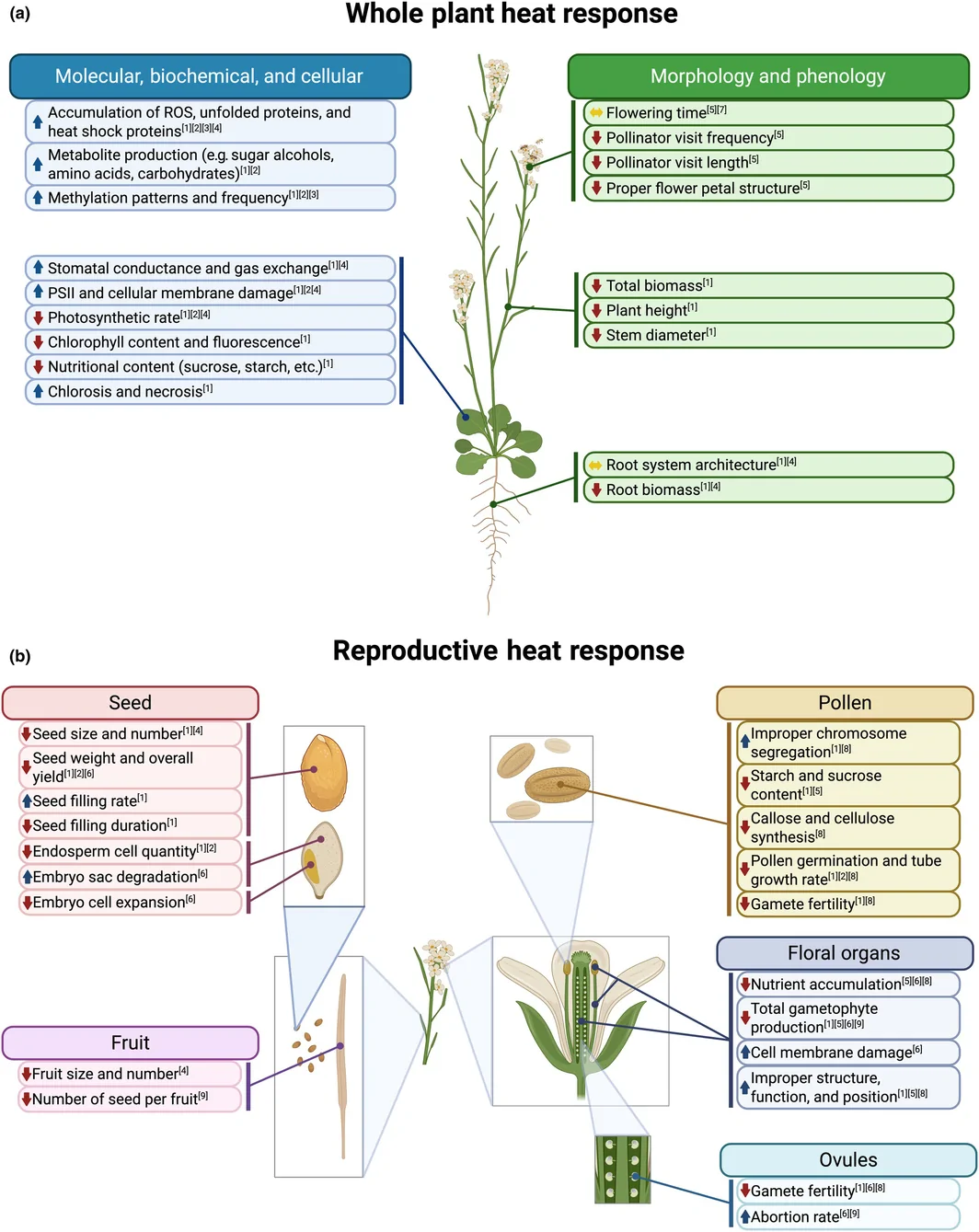
Generalized plant responses to heat stress across whole‐plant and reproductive scales. (a) Heat stress can impose a variety of effects on the whole plant by molecular, cellular, or physiological mechanisms. (b) Within reproductive tissues, heat stress is generally detrimental to production of gametes, fertilization, and seed production. Phenotypic increases are indicated with a blue arrow, decreasing with a red arrow, or variable changes with a yellow arrow. Superscripts represent references: [1] Chaudhary *et al*. (2020); [2] Kan *et al*. (2023); [3] Haider *et al*. (2021); [4] Jagadish *et al*. (2021); [5] Walters *et al*. (2022); [6] Wang *et al*. (2021); [7] Sinha *et al*. (2021); [8] Chaturvedi *et al*. (2021); [9] Ali & Muday (2024). Panels (a) and (b) were created in BioRender (BioRender.com/h3lv83e; BioRender.com/3z7svh1).

Sexual reproduction is a complex process that can fail at multiple stages under even mild heat stress (Zinn *et al*., 2010). Extensive research on food crops and model species has begun to elucidate the mechanisms by which heat disrupts reproductive processes (Fig. 1). These research efforts have led to the identification of heat‐tolerant crop varieties (Chaudhary *et al*., 2020), and gene families involved in heat‐stress responses (Tiwari *et al*., 2022; Kan *et al*., 2023). However, crop and model systems are not always representative of natural plant populations, particularly perennials and rare species (Kooyers *et al*., 2025a), suggesting that studying reproductive development in natural systems may reveal additional mechanisms and sources of variation. Indeed, crop scientists have increasingly recognized natural populations as valuable systems for identifying traits that enhance reproductive resilience to heat stress (Phillips *et al*., 2025). Below, we describe some of the key mechanisms of reproductive development identified through crop research.

Pollen development is often regarded as the most heat‐sensitive stage of reproduction (Zinn *et al*., 2010). Although the mechanistic basis for this sensitivity is not fully understood, several recurring patterns have been documented across plant species. For example, nutritive tapetal tissue within the anther can break down rapidly when exposed to heat during microsporogenesis, leading to pollen inviability (Chaturvedi *et al*., 2021). Additionally, heat stress can disrupt meiosis and proper chromosomal segregation (Bomblies *et al*., 2015; De Jaeger‐Braet & Schnittger, 2024; Khaitova *et al*., 2024; Lohani *et al*., 2025), thereby reducing the number of viable microspores. These developmental failures are rarely visible in common ecological fitness proxies such as plant size and flower number, yet they can substantially reduce reproductive success by constraining fertilization and seed set.

Ovule development is also sensitive to heat stress (Sage *et al*., 2015; Jagadish, 2020), although considerably fewer studies have explored the effects of elevated temperatures on the pistil (Wang *et al*., 2021; Resentini *et al*., 2023). Consequently, our understanding of heat‐induced ovule dysfunction is largely derived from a limited number of studies on crop systems (Wang *et al*., 2021). These studies found that heat exposure can cause abnormal embryo sac development (Shi *et al*., 2022), reduced ovule viability (Djanaguiraman *et al*., 2018), and increased sterility through excessive callose deposition in the ovary (Zhang *et al*., 2018). As with pollen, these impairments may occur despite apparently normal pistil morphology, allowing female reproductive failure to remain undetected in ecological assessments based on flower or fruit counts alone.

Fertilization and embryogenesis are also sensitive to heat (Sankaranarayanan *et al*., 2020). Heat exposure can accelerate embryo development, leading to a mismatch between seed coat and endosperm production and, ultimately, seed abortion (Mácová *et al*., 2022). Crucially, seed development could remain impaired even under favorable conditions if plants experienced elevated temperatures during earlier reproductive stages (Cope *et al*., 2023; Resentini *et al*., 2023; Tushabe *et al*., 2023; Kooyers *et al*., 2025b). Without testing seed viability, we may overestimate fitness when collecting fruit or seed counts.

Despite these reproductive sensitivities, the consequences of inviable pollen, dysfunctional ovules, or aborted seeds can go undetected when fitness is assessed using metrics such as flowering time, flowering duration, flower number (Gaudinier & Blackman, 2020), or fecundity measures such as fruit number (Buckley *et al*., 2021; Tushabe *et al*., 2025). To better understand how climate change shapes plant performance and evolutionary trajectories, ecological and evolutionary studies should explicitly account for the effects of elevated temperatures on pollen, ovule, and seed development. There are many well‐established methods for quantifying the impacts of elevated temperatures on plant reproduction. These methods include pollen viability assessments using staining techniques (Althiab‐Almasaud *et al*., 2024; Stokes & Geitmann, 2025) and profiling seed metabolites (Chebrolu *et al*., 2016). Notably, a recent handbook of plant reproductive trait measurements provides a particularly valuable resource with detailed protocols that can enable more direct and consistent quantification of reproductive responses across studies (Poschlod *et al*., 2026). Nonetheless, researchers should use caution when considering the type of stain and its application so as to not overestimate fitness (Stokes & Geitmann, 2025). Careful selection of traits and standardization of measurement approaches will be critical for understanding climate change across biological scales.

## Whole plant mechanisms affecting reproduction

While heat can directly disrupt gametophyte and seed development (Fig. 1), these processes are also vulnerable to physiological shifts that occur in other parts of the plant in response to elevated temperatures. For example, leaf exposure to heat can reduce sucrose and other important metabolites supplied to developing reproductive structures, resulting in the failure of pollen development (Santiago *et al*., 2021), or an imbalance in hormone signaling molecules can prevent successful fertilization (Sankaranarayanan *et al*., 2020; Ali & Muday, 2024). Ecological and evolutionary studies have not systematically integrated this whole‐plant perspective, but it is critical for understanding how heat affects reproduction. In this section, we briefly highlight some of the whole‐plant mechanisms involved in heat stress and their downstream effects on reproductive development (Fig. 1).

Heat stress can affect whole plant physiology through oxidative stress and hormonal changes. Heat induces both systemic oxidative stress and localized reactive oxygen species (ROS) accumulation in rapidly developing sink tissues, including floral organs. While moderate increases in ROS throughout the plant can promote stress tolerance (Huang *et al*., 2019; Mittler *et al*., 2022), excessive ROS accumulation in the flower can cause early tapetum degradation in the anther (Santiago *et al*., 2021), has been shown to reduce pollen production and viability (Lohani *et al*., 2025), and is associated with suppressed pollen tube growth and reduced fertilization efficiency (Wang *et al*., 2021; Ali & Muday, 2024). Hormonal disruption can further contribute to reproductive failure. Auxin is essential for anther and pollen development, and elevated temperatures may reduce auxin (Ozga *et al*., 2017), leading to reduced pollen viability and sterility even under mild heat stress (Chaturvedi *et al*., 2021; Jing *et al*., 2023). Mild heat stress can also induce ethylene production in leaves, which interacts with auxin and ROS pathways (Huang *et al*., 2023) and can affect reproductive development by promoting premature fruit senescence or delaying ripening depending on timing and tissue (Savada *et al*., 2017).

Beyond these molecular and hormonal effects, heat stress alters broader physiological processes including carbon assimilation and source–sink dynamics. Although photosynthesis is sensitive to elevated temperatures, plants can buffer against the effects of heat through stomatal regulation to maintain carbon assimilation over short time scales (Dusenge *et al*., 2019; Kan *et al*., 2023). By contrast, source–sink dynamics are often disrupted at lower temperature thresholds than those required to impair photosynthesis (Soltani *et al*., 2019). Mild heat stress can impair the allocation of nonstructural carbon into sugars and starch, potentially creating a net carbon deficit despite ongoing photosynthetic activity (Fatichi *et al*., 2014; Du *et al*., 2020). Because floral structures function as strong resource sinks (Santiago *et al*., 2021; Shen *et al*., 2023), disturbances to the source–sink dynamics during reproduction can have pronounced consequences for reproductive success. Altered carbon allocation has been linked to pollen sterility, seed abortion (Liu *et al*., 2021), and reduced seed production (Miret *et al*., 2024). Heat exposure has also been shown to reduce seed size, weight, number, and overall seed quality, reflecting both reduced carbon assimilation and impaired allocation to developing fruits during seed filling (Niu *et al*., 2021; Wang *et al*., 2021; Resentini *et al*., 2023).

Disruption of signaling pathways and source–sink mechanisms by elevated temperatures has significant implications for reproduction, yet these dynamics are not consistently incorporated into ecological and evolutionary studies. For example, meta‐analyses have identified correlations between biomass, warming, and fruit production (e.g. Younginger *et al*., 2017; Dobson & Zarnetske, 2025), which may be interpreted as evidence that growth traits serve as reliable proxies for reproductive output. Additionally, some studies utilize floral counts alone as proxies for reproductive fitness, which may not capture successful fertilization or seed development. Indeed, studies have shown that even short‐term heat exposure can drastically reduce pollen viability, seed set, and seed viability even when flower production is maintained under the same conditions (Notarnicola *et al*., 2021, 2022; Lohani *et al*., 2022, 2025; Zi *et al*., 2023). Similarly, ecological studies that focus solely on the heat tolerance of already‐formed pollen grains may miss the influence of whole‐plant physiological processes underlying pollen production. As a result, excluding whole‐plant physiological responses from reproductive development may lead to overestimates of fitness and inaccurate predictions of population persistence under climate change.

## Integrating reproductive development into ecological and evolutionary research

For decades, plant ecologists have investigated the effects of elevated temperatures on plant populations, often focusing on shifts in phenology (Price & Waser, 1998; Parmesan, 2006; CaraDonna *et al*., 2014; Inouye, 2020), altered patterns of selection (Etterson, 2004; Colautti & Barrett, 2013; Franks *et al*., 2018; Vtipil & Sheth, 2020; Santangelo *et al*., 2022; Anderson *et al*., 2025), and phenotypic plasticity (Atkin *et al*., 2006; Chevin *et al*., 2010; Nicotra *et al*., 2010; Arnold *et al*., 2022). These studies have documented global trends such as shifting flowering times (Parmesan, 2006), altered life‐history strategies (Boyko *et al*., 2023), conferred stress resistance through transgenerational plasticity (Donelson *et al*., 2018), and the potential for adaptive evolution (Anderson *et al*., 2025; Kooyers *et al*., 2025b). However, the extent to which elevated temperatures disrupt pollen development, and seed filling in natural populations remains poorly quantified, despite the high likelihood that these processes play a major role in shaping responses to future climates. Many key questions remain regarding both mechanistic and population responses to heat (Table 2). Now is the time for ecological and evolutionary studies to incorporate our understanding of the most sensitive life‐history stages into future research.

**Table 2:** Critical questions in heat‐induced reproductive failure: many questions remain regarding the genetic mechanisms, thermal limits, and variation underlying reproductive failure in heat stress, as well as the broader implications of these failures, and addressing these questions can enable researchers to bridge some of these knowledge gaps.

**Physiological and molecular mechanisms**
Why are certain conditions, such as elevated nighttime temperatures or altered diurnal regimes, particularly detrimental to reproductive processes? What is the relative importance of direct cellular damage (e.g. meiotic disruption, tapetal dysfunction) vs indirect physiological constraints (e.g. source–sink dynamics, sucrose transport) for reproductive failure under heat stress?
**Thermal limits**
What are the critical thermal thresholds for successful reproduction across species and populations? Can phenological shifts keep pace with increasing frequency of heat extremes, or will thermal mismatches persist?
**Variation, adaptation, and life history**
How much standing genetic variation exists for reproductive heat tolerance within and among natural populations? How do differences in reproductive biology and life history strategy (e.g. male vs female function, annual vs perennial strategies, taxonomic groups) influence vulnerability to heat?
**Population dynamics**
When are commonly used fitness proxies systematically overestimating reproductive success under heat stress? To what extent does reproductive failure under heat stress scale up to influence population dynamics and extinction risk?

First and foremost, it is crucial for ecologists and evolutionary biologists to recognize the critical vulnerability of plant reproduction to elevated temperatures. While many studies have focused on the effects of heat on photosynthesis, reproductive processes will be impaired at lower temperature thresholds than photosynthesis (Soltani *et al*., 2019; Liu *et al*., 2021). Characterizing the temperature ranges over which populations can successfully reproduce will be necessary to predict extinction risk under future climate change. For example, incorporating temperature dependence of the reproductive niche can improve species distribution modeling (Bykova *et al*., 2012). Further, demographic studies of climate change effects on plant populations highlight the importance of capturing reproductive thermal sensitivity for accurately forecasting population responses (Anderson *et al*., 2025). Greater clarity about future geographic distributions and population dynamics could be achieved by parameterizing models with reproductive thermal performance curves, including thresholds for pollen viability, fertilization success, and seed development. By integrating these reproductive limits with climate projections of mean and extreme temperatures, we can better assess population vulnerability under future scenarios.

Another crucial aspect of this research will be the intersection of phenology with reproductive responses to heat stress. Many natural plant populations already flower during cooler periods to avoid the negative impacts of high temperatures on reproduction (Luo *et al*., 2025). Future shifts in phenology through plasticity and evolution are expected, and these shifts may protect populations from increasing temperatures. However, such phenological shifts may not be sufficient to avoid episodic heat waves or overall warming temperatures. Consequently, populations will differ in their sensitivity to heat, reflected in the likelihood of reproductive failure. Those populations will also vary in their capacity to maintain successful reproduction under stress. These differences are likely to shape evolutionary responses to warming. Selection may favor genotypes with greater reproductive resilience to heat, whereas populations lacking sufficient standing genetic variation may experience recurrent reproductive failure, which may lead to population decline and local extinction.

While crop scientists now broadly recognize the deleterious effects of elevated temperatures on reproduction, the mechanisms underlying this failure are still not fully understood. Natural populations present an opportunity to identify the underlying mechanisms, an important goal for both conservation and improvement of crop resilience. In particular, natural systems with short generation times and well‐established genetic resources provide a powerful framework for identification of the ultimate causes of reproductive failure under elevated temperatures, along with identifying the genes harboring standing genetic variation for resilience (Kooyers *et al*., 2025a, b). Studies of such systems could clarify the relative contributions of source–sink dynamics, meiotic disruption, tapetal dysfunction, and other processes that influence pollen vulnerability. Further, studies in these systems could uncover why nighttime temperatures are so critical for successful pollen formation and seed filling. Elevated nighttime temperatures have reduced yield in several major crops, including soybean, rice, maize, and wheat (Sakai *et al*., 2022; Giménez *et al*., 2025; Thenveettil *et al*., 2025), but the mechanisms by which nighttime heat affects reproductive development remain poorly understood. Nighttime temperatures are increasing at a higher rate than daytime temperatures, causing the daily temperature range to shrink, especially in the Northern Hemisphere (Sun *et al*., 2018). Decreasing daily thermal ranges can have profound effects on organisms but remain largely unexplored (Carter & Fleming, 2025). Studies incorporating nighttime warming and its effects on reproductive development will provide valuable insight for ecological and evolutionary research while also informing crop improvement efforts.

Going forward, studies of warming climate impacts on natural populations should aim to quantify the effects of temperature on reproduction. While fully dissecting the underlying physiological mechanisms may be impractical for many species in natural plant communities, quantification of pollen and seed responses to elevated temperatures can be highly informative for documenting the impacts of heat stress on reproduction. Although a thorough discussion of quantification techniques is beyond the scope of this piece, several recent publications review assays and measurements which can be utilized during controlled temperature manipulations in the field or glasshouse to inform our predictions of how natural populations will be impacted by warming (e.g. Stokes & Geitmann, 2025; Auroux *et al*., 2026; Poschlod *et al*., 2026). For systems amenable to experimental manipulation, evolutionary biologists should aim to quantify phenotypic and genetic variation in reproductive tolerance to elevated temperatures, identify the underlying, allelically variable genes, and model how natural populations will respond evolutionarily through gene flow, genetic drift, and natural selection. While these tasks may be challenging to accomplish, research incorporating any of these approaches or addressing key questions (Table 2) will provide a more complete understanding of the impacts of heat on plant populations and inform strategies to preserve biodiversity and further the iterative process of research and discovery.

## Competing interests

None declared.

## Author contributions

DAD, ALT, EBJ, JHW and DBL conceived of the project, drafted, revised, and approved the manuscript. DAD and ALT contributed equally to this work.

## Disclaimer

The New Phytologist Foundation remains neutral with regard to jurisdictional claims in maps and in any institutional affiliations.
